# Virtual Reality Simulation for Assessment of Hemorrhage Control and SALT Triage Performance: A Comparison of Prehospital to In-Hospital Emergency Responders

**DOI:** 10.1017/S1049023X25101349

**Published:** 2025-08

**Authors:** Nicholas Kman, David Way, Ashish R. Panchal, Jeremy Patterson, Jillian McGrath, Douglas Danforth, Ashutosh Mani, Dave Babbitt, Jacob Hyde, Brian Pippin, Ewart de Visser, Jennifer McVay

**Affiliations:** 1. The Ohio State University, Columbus, Ohio USA; 2. Big Bear AI, Columbia, Maryland USA; 3. Warfighter Consulting, Scottsdale, Arizona USA; 4. CACI, Inc, Falls Church, Virginia USA; 5. De Visser Research, Springfield, Virginia USA

**Keywords:** disaster planning, Emergency Medical Services, emergency responders, hemorrhage control, mass-casualty incidents, triage, virtual reality, educational

## Abstract

**Introduction:**

Targeted identification, effective triage, and rapid hemorrhage control are essential for optimal outcomes of mass-casualty incidents (MCIs). An important aspect of Emergency Medical Service (EMS) care is field triage, but this skill is difficult to teach, assess, and research.

**Study Objective:**

This study assessed triage efficacy and hemorrhage control of emergency responders from different professions who used the Sort, Assess, Life-Saving Treatment (SALT) triage algorithm in a virtual reality (VR) simulation of a terrorist subway bombing.

**Methods:**

After a brief just-in-time training session on the SALT triage algorithm, participants applied this learning in First *VR*esponder, a high-fidelity VR simulator (Tactical Triage Technologies, LLC; Powell, Ohio USA). Participants encountered eleven virtual patients in a virtual scene of a subway station that had experienced an explosion. Patients represented individuals with injuries of varying severity. Metrics assessed included triage accuracy and treatment efficiency, including time to control life-threatening hemorrhage. Independent Mann-Whitney analyses were used to compare two professional groups on key performance variables.

**Results:**

The study assessed 282 participants from the ranks of EMS clinicians and medical trainees. Most (94%) participants correctly executed both global SALT sort commands. Participants triaged and treated the entire scene in a mean time of 7.8 decimal minutes, (95%CI, 7.6-8.1; SD = 1.9 decimal minutes) with a patient triage accuracy rate of 75.8% (95%CI, 74.0-77.6; SD = 15.0%). Approximately three-quarters (77%) of participants successfully controlled all life-threatening hemorrhage, within a mean time of 5.3 decimal minutes (95%CI, 5.1-5.5; SD = 1.7 decimal minutes). Mean time to hemorrhage control per patient was 0.349 decimal minutes (SD = 0.349 decimal minutes). Overall, EMS clinicians were more accurate with triage (P ≤ .001) and were faster at triage, total hemorrhage control (P < .01), and hemorrhage control per patient (P < .004) than medical trainees.

**Conclusions:**

Through assessments using VR simulation, it was observed that more experienced individuals from the paramedic (PM) workforce out-performed less experienced medical trainees. The study also observed that the medical trainees performed acceptably, even though their only formal training in SALT triage was a 30-minute, just-in-time lecture. Both of these findings are important for establishing evidence that VR can serve as a valid platform for assessing the complex skills of triage and treatment of an MCI, including the assessment of rapid hemorrhage control.

## Introduction

The proliferation of mass-casualty incidents (MCIs) in the United States is becoming a major challenge.^1,2^ Well-prepared first responders, trained to identify and prioritize potential survivors, contribute to maximizing lives and limbs saved.^3^ Unfortunately, contemporary training methods, which include lectures, tabletop exercises, or live-action drills, have been found deficient.^2^

Current triage training methods typically require trainees to read and review online learning modules with images and case descriptions.^4^ These modules fail to provide hands-on training in an environment resembling that of an MCI, lacking the realism, stress, and chaos that prevents first responders from being able to transfer their triage knowledge and skills to the actual emergency event. In contrast, virtual reality (VR) simulation provides available and affordable immersive and realistic visual environments and auditory soundscapes for first responder training.^5,6^ These simulations allow first responders to practice the steps of triage directly and interactively in an environment more accurately resembling a real MCI. These systems can be used to provide first responders with deliberate practice, training them to mastery levels of performance.^7^ Simulation through VR can further provide realistic and dynamic responses after changes in treatment or resources, a feature which is not available in most drills, tabletop exercises, or retrospective review studies.^8^ The system can also record the performances of the trainee and generate reports that can be used to provide detailed feedback after the simulated encounter.^9^

Akin to the lack of training opportunities for first responders, there is also a dearth of opportunities to research and evaluate different triage protocols.^10^ A relatively new triage protocol, Sort, Assess, Life-Saving Treatment and Transport or (SALT) triage, has been proposed and adopted as a standard by numerous professional organizations across the United States. The SALT triage method represents a logical, evidence-based algorithm that includes life-saving interventions, a new “expectant” category, and pediatric integration, making it a good option for mass-casualty triage.^11^

The purpose of this study was to demonstrate the use of a VR simulation to train and assess participants from two professions on their SALT triage and treatment performances. To this end, this study leveraged First *VR*esponder (V1; Tactical Triage Technologies, LLC; Powell, Ohio USA), a novel VR simulation of a terrorist subway bombing, to train and assess Emergency Medical Service (EMS) professionals, along with emergency medicine trainees (EM).^6^ The hypothesis was that EMS clinicians would out-perform EM trainees due to their training and experience in the prehospital setting. Using this framework, SALT triage and treatment efficiency and accuracy were assessed.

## Methods

### Population of Interest and Sampling

This is a prospective investigation designed to compare the triage and treatment performance of individuals from two professions that differ on training and experience with MCIs. To that end, a VR assessment platform that was designed to present a standardized and realistic MCI scenario to emergency responders was used for this study. The computerized VR assessment platform not only presented the stimulus for assessment, but it was also designed to gather and record all performance metrics related to triage and treatment of the MCI event. Over the past two-and-a-half years, the First *VR*esponder system has been used to deliver SALT triage training and performance assessments to individuals from a variety of agencies throughout one midwestern state in the United States. All data gathered from encounters with the First *VR*esponder simulator were transferred and stored in a secured data registry, which was established to serve as an active laboratory for research on triage and treatment of MCIs. First *VR*esponder and this research was initially funded under grant number R18HS025915 from the Agency for Healthcare Research and Quality (AHRQ; Rockville, Maryland USA).^6^ To date, the registry holds triage and treatment performance data generated from nearly 700 individuals, including EMS clinicians, medical professionals and trainees, nurses, public health officials, military medics, and others who have participated in the VR simulation encounters. All participants provided informed consent for the use of their performance data for research.

For this study, the recorded performances of a subset of 282 participants from the registry whose VR encounters involved implementing SALT triage in a moderately challenging scenario with a standard set of eleven virtual patients were selected. These data were used to evaluate how participants from different professions performed on fundamental triage metrics including hemorrhage control and triage accuracy after receiving just-in-time SALT triage training. For this study, participants included 210 EMS clinicians (191 paramedics [PMs] and 19 emergency medical technicians [EMTs]) along with 72 medical trainees (32 EM residents [RES] and 40 senior medical students [MS]). The number of participants in each sample group was proportional to the populations of specialists from these groups throughout the local region. Additionally, because of unequal groups and measures that were not normally distributed, non-parametric statistics were used for analyses as indicated below. This study was approved by The Ohio State University Institutional Review Board (Columbus, Ohio USA; IRB Protocol # 2020B0128).

### First VResponder Simulation

**Figure 1.**
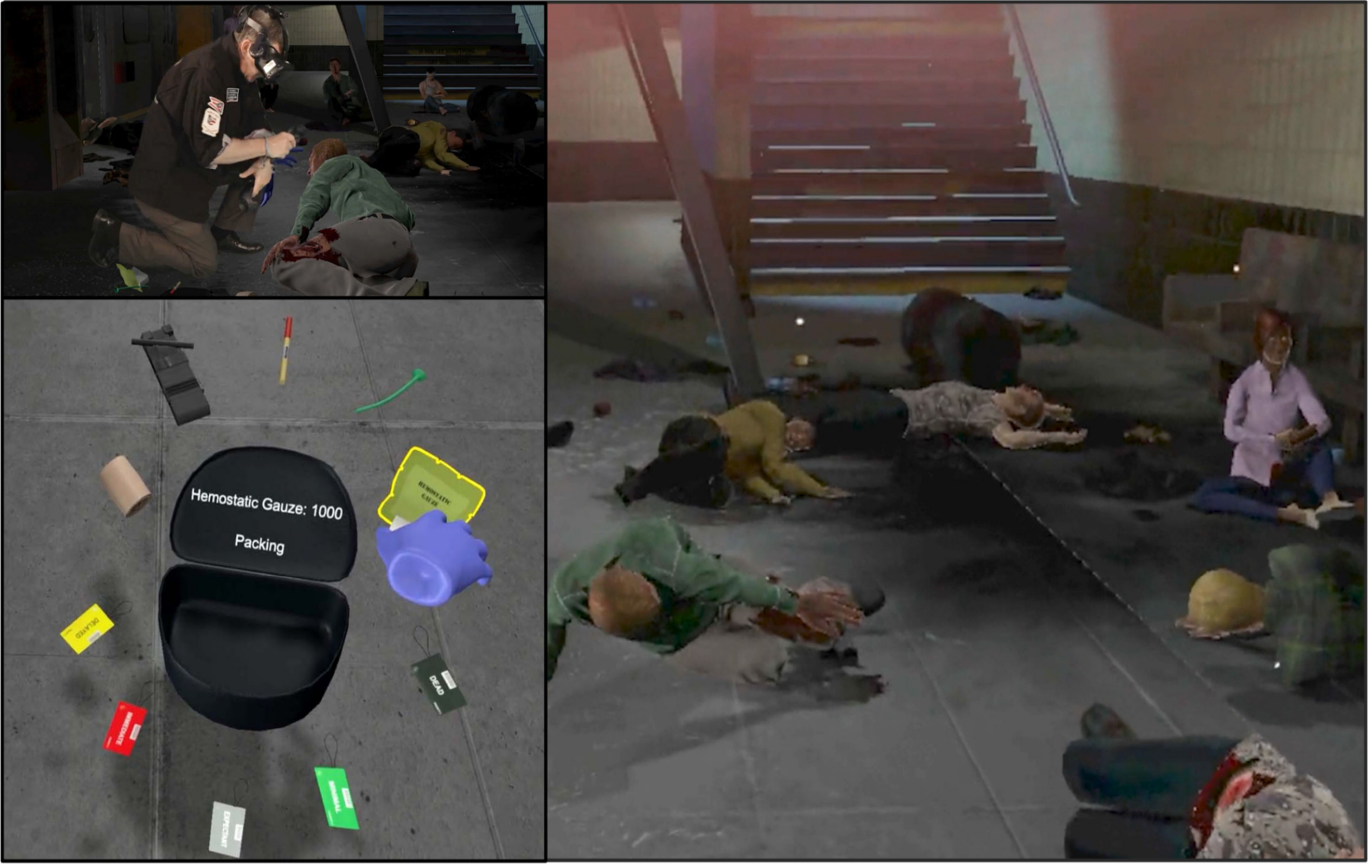
The First VResponder Simulation Experience. Note: Participants don a virtual reality (VR) head-mounted display (HMD) and encounter patients within a subway bombing scenario (right). Participants use their voice and hand-held controllers to interact with a VR system to diagnose and treat patients (upper left). Participants have a medical supply bag available allowing them to choose different interventions for treating patients (lower left).

The First *VR*esponder simulation has been described elsewhere but will briefly be summarized here.^6,12,13^ The simulation was designed with Unity Pro (Unity Software, Inc; San Francisco, California USA), a software platform for developing video games using three-dimensional graphics (Figure 1). The program is interactive, fully immersive, and automated. It runs on a commercially available laptop gaming computer with a Meta Quest 2 VR headset (Reality Labs, Meta Platforms; Menlo Park, California USA). The layout for these training sessions involved eleven virtual patients with a variety of life-threatening (eg, acute arterial bleeding, penetrating trauma, pneumothorax, amputations, or burns) and non-life-threatening (eg, superficial lacerations, psychological distress, confusion, or ruptured ear drums) injuries.

### Study Procedure

Training for SALT triage took the form of a just-in-time brief didactic session that explained how the protocol worked and how it was different from other triage protocols.^14^ The training was delivered to participants just prior to their individual encounter with the First *VR*esponder, system (Figure 1). Participants then completed a four-patient practice tutorial using the system, before encountering the full-scale VR scenario involving a subway station explosion resulting in eleven patients with various states of injury. During their encounter, each participant’s performance data were recorded and saved.^6,12^

After completion of the encounter, participants were debriefed on their performance with an experienced EMS educator guided by system-generated reports of participant performance. Participants took an average of 30 minutes to complete all aspects of the session. First *VR*esponder participants perceived the encounter as realistic and effective for training, regardless of their prior level of training or their experience using VR.^9^

### Outcome Measures

Using the comparisons of performances of individuals from different professions, the study’s primary focus was on SALT triage and treatment efficiency and accuracy. The First *VR*esponder simulation system recorded participants’ performance in a detailed, time-stamped data log. From this log, metrics were extracted to evaluate performance, including triage efficiency and accuracy. Key efficiency metrics, defined as critical measures by others, included time to triage the scene, hemorrhage control for all life-threatening bleeding, and time to hemorrhage control per patient^15^ (Appendix 1; available online only). After defining key metrics such as “life-saving treatments” and “triage categories,” a content expert panel of emergency physicians with disaster medicine experience (NEK, ARP, JM) independently coded what they believed were the correct “answers” for each virtual patient case in the library of virtual patients. There are currently 20 patient cases in the library, eleven of which were used for this project. Panelists looked at each victim or patient in the scenario and defined key metrics such as needed “life-saving intervention” and “triage category.” The team deliberated correct answers for each patient case through an iterative process of scoring and revising until consensus was reached. The final scoring algorithm was programmed into the system and was used to score participant performance. The initial scoring algorithms were adjusted over time based on feedback received from prehospital participants. Overall, SALT triage performance was calculated using a count and percentage of correctly categorized virtual patients. Definitions for triage errors (over-triage and under-triage) were adopted from a previous study.^16^

Based on the SALT protocol, the expert panel also defined an “ideal” sequence in which the eleven patients should be attended to by first responders to minimize loss of life or limb. A value was calculated to define how close participants were to the ideal order by counting the number of patients who were not attended to in the ideal order. This metric was called “SALT Sequence Adherence Performance.” The SALT Sequence Adherence Performance score is flexible, allowing for variation in performance based on proximity to patients and other variables.

### Data Analysis

Data were obtained from the registry by filtering, parsing, and extracting time-stamped, individual performance logs into a de-identified database formatted for analysis. All triage efficiency measures were originally extracted in milliseconds and converted to a decimal format of minutes and seconds for analysis. In some instances, time was converted to standard minutes and seconds format for reporting. Descriptive statistics were calculated for all the triage efficiency measures including mean, standard deviation (SD), median, and interquartile range (IQR). Percentages were also calculated for the triage performance metrics.

Since the measures of triage and treatment performance, described above, were not normally distributed, they were treated as ordinal level data and analyzed using independent samples Mann-Whitney tests. This analytic approach required Bonferroni corrections for multiple comparisons to control Type 1 error rates (a common problem that occurs when conducting multiple statistical comparisons). The independent variable was professional group (EMS clinicians versus medical trainees). The study (family)-wise P value for statistical significance was set at .05. Since Mann-Whitney comparisons between groups were being made across eight measures, the adjusted P value for statistical significance was set at P <.005 (.05/9). The Mann-Whitney tests were supplemented with Cohen’s D effect sizes and graphics to aid in the interpretation of observed differences.^17,18^

## Results

Participant’s prior experience with SALT triage training was estimated through an analysis of the entire data registry, which suggested that prior to their First *VR*esponder encounter, only one-third of participants in this study had received SALT triage training. Overall, EMS clinicians (75%) were more likely than medical trainees (35%) to have received some form of triage training other than SALT.^9^

### Triage Performance

Nearly all participants effectively issued the global sort commands walk and wave (93.6%; 264 of 282) with no difference between professional groups (Table 1).

**Table 1.** Comparing SALT Triage Performance of 72 Medical Trainees (MS and RES) with 210 EMS Clinicians (EMTs and PMs) using Independent Samples Mann-Whitney U and Chi Square Tests on Key Variables in Triage of VR MCI Involving Eleven Patient Victims

Triage Performance	Group	N	Freq	Pct			*X* ^2^	P	ES*
SALT Adherence: Global Sort Commands (Walk & Wave)	Medical	72	66	91.7			.615	.413	NA
	EMS	210	198	94.3					
	Total	282	264	93.6					
SALT Sequence Adherence Performance: (Swaps to Perfect Order)	**Group**	**N**	**Mn**	**SD**	**95% CI**	**Mn Rnk**	**MW-U**	**P**	ES*
	Medical	72	1.3	0.90	1.1-1.5	135	8,006	.423	NA
	EMS	210	1.4	1.12	1.3-1.6	144			
	Total	282	1.4	1.1	1.3-1.5				
Triage Tag Accuracy	Medical	72	69.1	16.4	65.2-73.0	106	10,098	<.001	.523
	EMS	210	78.1	13.8	76.3-80.0	154			
	Total	282	75.8	15.0	74.1-77.6				
Triage Accuracy: Over-Triage Errors	Medical	72	13.8	10.1	11.4-16.1	174	5,214	<.001	.481
	EMS	210	8.7	9.4	7.5-10.0	130			
	Total	282	10.2	9.8	8.9-11.2				
Triage Accuracy: Under-Triage Errors	Medical	72	8.7	11.6	6.0-11.4	137	7,895	.555	NA
	EMS	210	8.6	9.0	7.4-9.9	143			
	Total	282	8.7	9.7	7.5-9.8				
Triage Accuracy: Critical Triage Errors	Medical	72	5.0	7.2	3.3-5.0	156	6,519	.032	NA
	EMS	210	3.0	5.2	2.3-3.7	137			
	Total	282	3.5	5.8	2.8-4.2				
Triage Efficiency: Time to Triage Scene in Decimal Minutes	Medical	72	8.7	1.8	8.3-9.2	184	4,483	<.001	.645
	EMS	210	7.5	1.8	7.3-7.8	127			
	Total	282	7.8	1.9	7.6-8.1				

Abbreviations: SALT, Sort, Assess, Life-Saving Treatment triage; MS, medical student; RES, emergency medicine resident; EMS, Emergency Medical Services; EMT, emergency medical technician; PM, paramedic; VR, virtual reality; MCI, mass-casualty incident.

Note: *ES = Cohen’s D Effect Sizes which are generally interpreted as: 0.0-0.1 = No effect; 0.2-0.4 = Small effect; 0.5-0.7 = Intermediate effect; >.80 = Large effect. NA = Effect sizes are not reported for non-significant statistical tests.^26^

**Figure 2.**
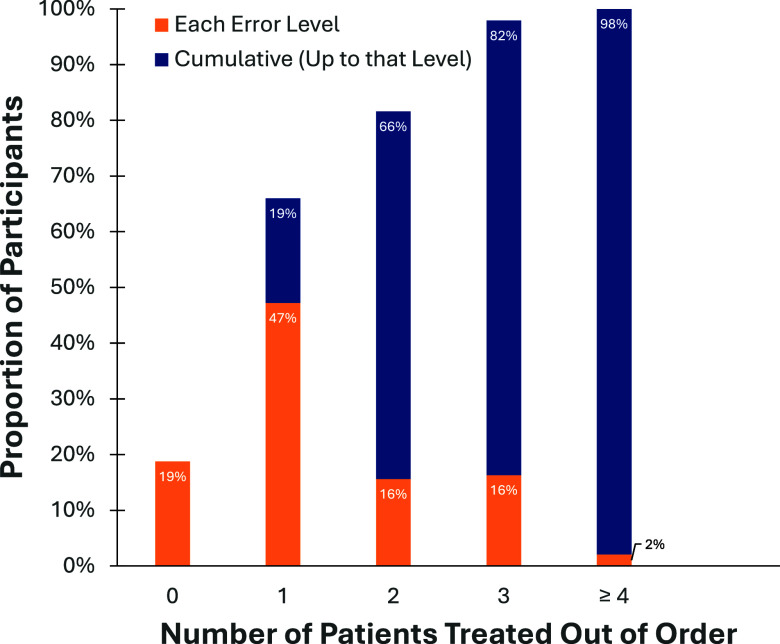
SALT Adherence Performance for All Responders. Note: The x-axis indicates the number of patients that were treated out of order (errors). Fewer patients treated out of order indicates better performance. The orange bars indicate the percentage of responders that performed at that level. The blue bars indicate the percentage of responders that performed at that level or below (cumulative).

Using the SALT Adherence Performance Metric, it was observed that adherence to the recommended sequence in which patients were seen was relatively high with 82% of responders executing the prescribed order within three or fewer errors (Figure 2). Perfect adherence to the prescribed patient treatment order was accomplished by 19%. A single patient was treated out of order by 47% and two patients were treated out of order by 16% (Figure 2). There was no significant difference between professional groups on SALT Adherence Performance (Table 1).

**Figure 3.**
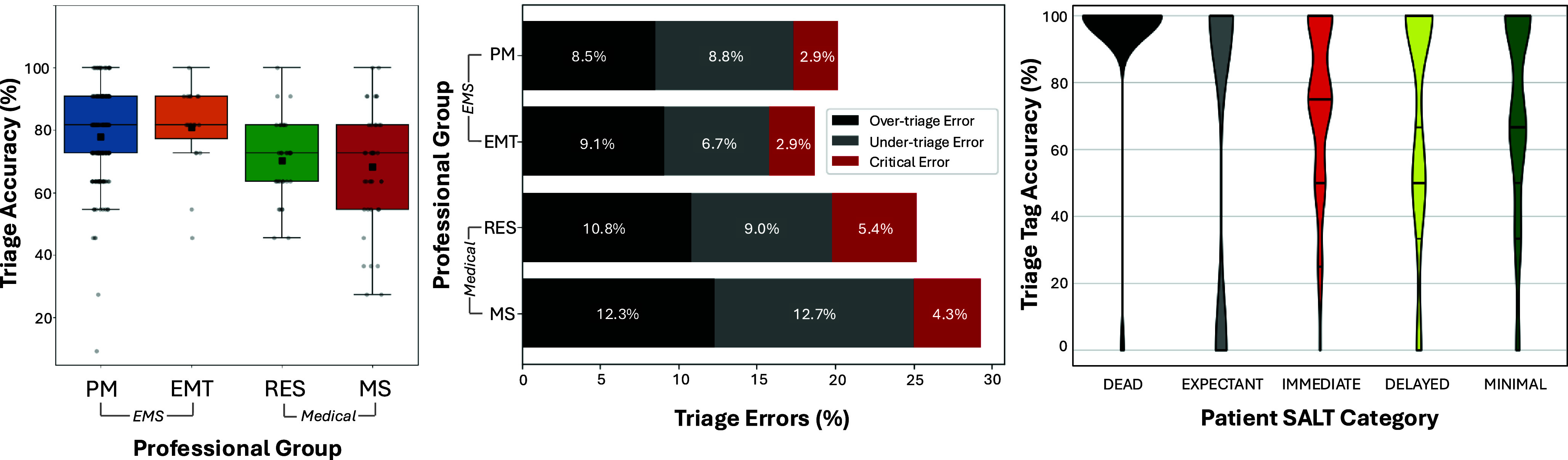
(a) Triage Accuracy by Group; (b) Triage Errors by Category and Group - Trial Errors Categorized as Over-Triage, Under-Triage, and Critical Errors; (c) Triage Accuracy by Tag. Abbreviations: PM, paramedic; EMT; emergency medical technician; RES, emergency medicine resident; MS, medical student.

The mean triage accuracy rate (or the percentage of patients accurately tagged) was 75.8% (95%CI, 74.1-77.8%; SD = 15%). A significant effect was observed for group on triage accuracy, *U* (2) = 10,098; *P <* .001; *d* = 0.52, suggesting that EMS clinicians had significantly higher triage accuracy than their medical counterparts. The associated effect size indicated that this was an intermediate or medium sized effect (Table 1 and Figure 3).

Participants’ overall over-triage error rate averaged 10% (95%CI, 8.9-11.2; SD = 9.8%). A significant effect was observed for group on over-triage error rate, *U* (2) = 5,214; *P <* .001; *d* = 0.48. Overall, EMS clinicians committed significantly fewer over-triage errors than medical trainees (Table 1 and Figure 3). The under-triage rate was 8.7% (95%CI, 7.5-9.8; SD = 9.7%) and the critical error rate was 3.5% (95%CI, 2.8-4.2; SD = 5.8%). No group differences were observed for under-triage and critical errors (Table 1 and Figure 3). Critical errors were likely to incur irrevocable patient morbidity or mortality and were most likely to occur when victims were placed erroneously into either “dead” or “expectant” categories.

While statistical comparisons were not made between groups on their triage accuracy by tag, the violin plot provided some hints about which triage categories responders found more challenging. Overall, responders had the lowest performance with “delayed” patients (*M* = 50%) and the highest performance with the “dead” patient (*M* = 96.1%). The “expectant” patient showed a dichotomous distribution pattern, which suggested a considerable number of responders were inaccurate in applying this tag (Figure 3).

### Triage Efficiency

**Figure 4.**
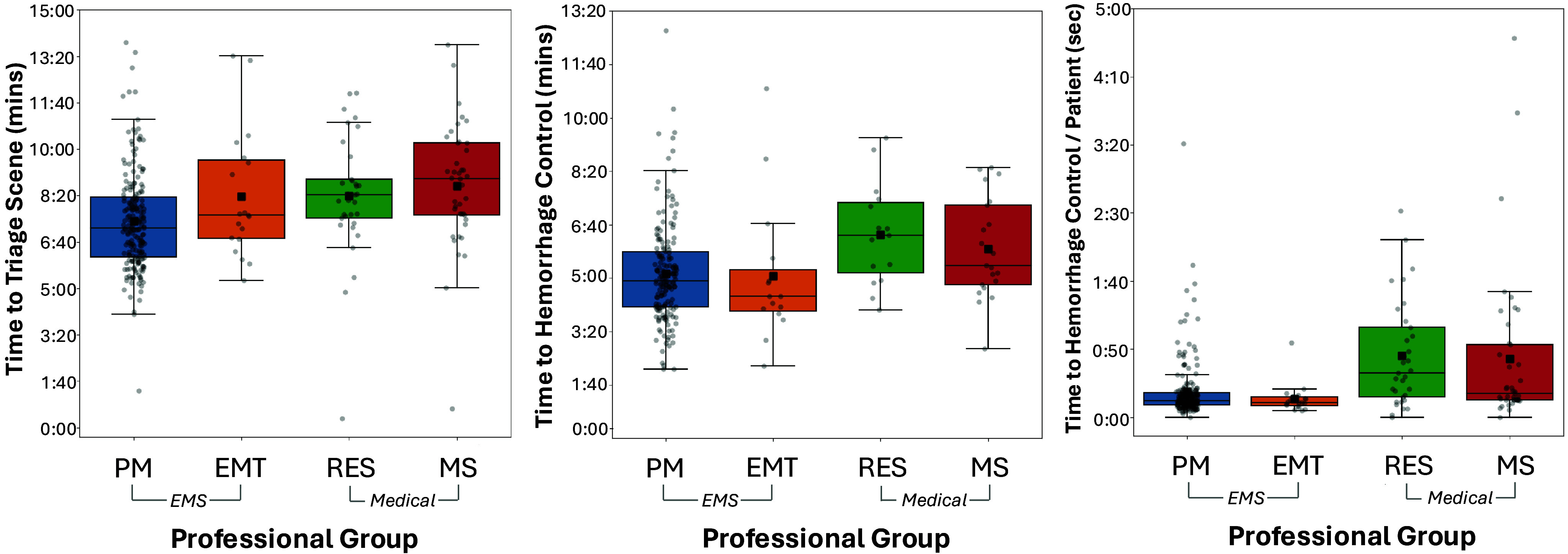
Time to Triage the Scene (left), Time to Hemorrhage Control (middle), and Time to Hemorrhage Control per Patient (right) by Group. Note: The line within the boxplot indicates the median and the black square indicates the mean. Abbreviations: PM, paramedic; EMT; emergency medical technician; RES, emergency medicine resident; MS, medical student.

The overall mean time to scene triage was 7.8 in decimal minutes (95%CI, 7.6-8.1; *SD* = 1.9). There was a significant effect for group on time to scene triage (*U* (2) = 29.04; *P ≤* .001; d = 0.64) with EMS clinicians triaging the scene faster than medical trainees (Table 1 and Figure 4).

A robust 77% of responders successfully controlled all life-threatening hemorrhage; EMS clinicians (87%) were significantly more likely than medical trainees (50%) to achieve hemorrhage control of all bleeding patients in the scene (*X* ^2^(1); P ≤ .001; d = 0.826). Among the N = 218 participants who controlled all life-threatening hemorrhage, the overall average time for time to hemorrhage control for all life-threatening bleeding was 5.3 decimal minutes (95%CI, 5.1-5.5; SD = 1.7). There was a significant effect for group on time to achieve hemorrhage control, *U* (2) = 2,027; *P ≤* .001; *d* = 0.505. Overall, EMS clinicians were faster to achieve hemorrhage control than medical trainees (Table 2 and Figure 4).

**Table 2.** Comparing Times to Total Scene and Per Patient Hemorrhage Control in VR Simulation of an MCI for 36 Medical Trainees (MS and RES) and 182 EMS Clinicians (EMTs and PMs) Using Independent Samples Mann-Whitney U Tests on Time to Reach Hemorrhage Control for Bleeding Injuries among Eleven Patient Victims

	Group	N	Mn	SD	95% CI	Mn Rnk	MW-U	P	ES*
Hemorrhage Control: Time to Control All Bleeding Injuries in Decimal Minutes†	Medical	36	6.1	1.6	5.6-6.6	144	2,027	<.001	.505
	EMS	182	5.1	1.6	4.9-5.4	103			
	Total	218	5.3	1.7	5.1-5.5				
Hemorrhage Control: Time to Per Patient Hemorrhage in Decimal Minutes†	Medical	36	0.55	0.69	0.31-0.78	144	2,271	.004	.402
	EMS	182	0.31	0.37	0.26-0.36	103			
	Total	218	0.35	0.45	0.29-0.41				

Note: Times are reported only for the 77% of subjects (218 of 282) who attained total hemorrhage control. † Only those who successfully treated all bleeding injuries are included.

Abbreviations: MS, medical student; RES, emergency medicine resident; EMS, Emergency Medical Services; EMT, emergency medical technician; PM, paramedic; VR, virtual reality; MCI, mass-casualty incident.

Note: *ES = Cohen’s D Effect Sizes which are generally interpreted as: 0.0-0.1 = No effect; 0.2-0.4 = Small effect; 0.5-0.7 = Intermediate effect; >.80 = Large effect. NA = Effect sizes are not reported for non-significant statistical tests.^26^

Participant’s average time for hemorrhage control per patient was 0.35 decimal minutes (95%CI, 0.29-0.41; SD = 0.35). There was a significant effect for group on time to achieve hemorrhage control per patient, *U* (2) = 11.5; *P =* .004; *d* = 0.402. Pairwise comparisons revealed that EMS clinicians were faster than medical trainees at achieving hemorrhage control on a per patient basis (Table 2 and Figure 4).

## Discussion

This work presents one of the largest studies to evaluate triage efficiency and accuracy as it pertains to first responders’ hemorrhage control of virtual patients in a VR simulation of an MCI. The observed triage accuracy of 75.8% and a hemorrhage control completion time of less than five minutes are encouraging, given the region’s new adoption of SALT triage as a standard for EMS clinicians. These measures align with results from other studies^19^ and, particularly given the realistic distractions, anxiety, and stress recreated in the VR simulation, demonstrate successful adoption of the SALT paradigm. Indeed, this study demonstrates the utility of an immersive, high-fidelity VR system for training and evaluating emergency response personnel.

An important additional finding was that participants from the pool of EMS clinicians out-performed novices from the pool of medical trainees on many of the chosen triage and treatment metrics. In measurement terms, this finding would be interpreted as a form of criterion-related validity because the results from the platform were able to confirm the hypothesis that scores would effectively discriminate between more experienced and less experienced participants.^18^ This finding further underscores the importance of experience and continuous practice in achieving higher levels of triage proficiency. Finally, it is important to note that while the more experienced EMS clinicians out-performed medical trainees, the medical trainees’ performance was acceptable considering that they had only one brief just-in-time didactic and one simple practice trial. Nearly 99% of medical trainees (RES and MS) had never experienced an actual MCI. Therefore, their observed performance scores were notably high given their lack of real-world experience. This observation is attributed primarily to the fact that these learners are highly motivated to learn about how to treat and triage an MCI. Additionally, all learners had some experience with seriously injured patients in the hospital setting and received a rather robust just-in-time lecture and practice session prior to their measured encounter with the VR system. This suggests that even novices can learn and execute triage effectively with minimal investments in quality training.

As a core aspect of clinical management in mass-casualty events, many reports have recommended that training must include hemorrhage control techniques on a variety of injury patterns, including the use of tourniquets, pressure dressings, and hemostatic wound packing.^19^ This novel simulation allowed for the tracking and assessment of hemorrhage control for patients in extremis and the effectiveness of rapid application of life-saving interventions, such as tourniquets, using SALT. Military medical studies have found that hemorrhage volumes and time to critical blood loss are associated with anatomical location of the bleeding. Consequently, depending on the area of the body, critical blood loss can occur within two-to-five minutes of uncontrolled hemorrhage onset.^14,20^ The providers in this study stayed within this range by achieving hemorrhage control in an eleven-victim scene in approximately five minutes and, when the provider recognized hemorrhage in the simulation, it was controlled in an average time of 21 (or 0.35 decimal) seconds per patient. These fast times may be attributed to SALT triage’s emphasis on the global sorting of patients,^11,13^ which allows for the swift location and treatment of patients who are not moving or have an obvious life threat, like hemorrhage.

Nearly all responders issued the global sort commands, even those who were considered novices. Furthermore, once the scene was sorted, it was observed that adherence to the SALT protocol was relatively high with 82% of responders executing the prescribed sequence with only two or fewer errors (Figure 2). The extremely efficient time to hemorrhage control was most likely explained by the effectiveness of sorting the scene to find the more seriously injured patients. Further comparison of SALT triage and other triage algorithms on hemorrhage control is currently being studied within this controlled VR environment.

Training with VR can also assist with discovering common errors and improving SALT training practices by identifying specific difficulties learners had within simulations. In the current study, learners struggled with the “expectant” category, which was one of the most inaccurately applied triage tags. The SALT triage method introduced this category, which responders apply based on their assessment of available resources. The “expectant” category is intended to be flexible and aids in identifying dying patients so that they can receive resuscitation or comfort care.^11,13^ Many subjects familiar with other triage algorithms that do not use “expectant,” like Simple Triage and Rapid Treatment (START), may have been confused or unsure of the application of this tag. To improve participant performance accuracy when triaging the “expectant” category, future training will use objective physiological markers to distinguish critical but potentially salvageable patients from those with un-survivable injuries. Examples of “expectant” patients include those with injuries such as massive head trauma with no purposeful movement; or patients with open skull fractures presenting with exposed brain matter along with agonal breathing (similar to the patient in the simulation). Additional examples could include severe burns covering >90% of total body surface area with no viable treatment options or no palpable central pulses despite initial interventions.

Another challenging triage category was the “delayed” category. The SALT triage method teaches responders to think of patient conditions as dynamic, suggesting for example, that if a tourniquet works to stop hemorrhage and sustain patient viability, then responders should categorize a patient “delayed” instead of “immediate.” Many subjects in this study (particularly the medical trainees) typically over-triaged patients by basing their patient assessment on their initial presentation instead of their condition after treatment.

## Limitations

There were several limitations to this study. First, adapting to the virtual environment requires time and experience. The participants’ performances in the VR environment may have been hindered by a broad lack of experience with using VR platforms. Consequently, time to triage the scene and treat hemorrhage control may be slightly inflated as participants learned to use controllers to navigate and function in the VR system. Second, in rare instances, the simulator failed to provide sufficient feedback to the participant that a treatment was incorrectly applied. This technical error occurred when a trainee didn’t apply the desired tool in proximity to the invisible collider on the patient. On these occasions, the participant would not receive credit for a treatment that didn’t hit the precise mark needed for detection by the computer system. This observation was temporarily mitigated by the moderator who provided real-time verbal feedback to the responder during their encounter. Eventually, programmers were able to enlarge colliders to prevent these problems.

Finally, the VR world is believed to have sufficient *psychological fidelity,* referring to how accurately the simulation evokes the same cognitive demands, decision-making processes, and psychological cues required to perform triage during a mass-casualty event.^21–23^ However, the simulation does not contain all the sensory experience of the real world, like the smell or feel of human injuries. Further, a subway bombing scenario may not be the type of MCI most first responders encounter. Even with these minor shortcomings, learners overwhelmingly expressed satisfaction at the level of realism and immersion with the benefits and potential for learning far outweighing the limitations.^9^

## Future Directions

Given the state of VR technology, the system approximates “real-world” conditions as closely as is possible. Plans for the immediate future include: accommodating more than one participant in the virtual environment at a time so as to provide the opportunity for teamwork training; monitoring physiological conditions of responders (such as heart rate) during their encounter so that there is a better understanding of situations that increase stress or anxiety; and finally, complementing the VR encounter with hands-on training of medical treatments, such as applying a tourniquet, on task trainers. Finally, as technology improves, haptic gloves, full body haptic suits, augmented reality supplements, and other changes can improve the psychological fidelity of these scenarios.

Currently, First *VR*esponder is customizable so that the level of difficulty of a scenario can be adjusted by: increasing the level of chaos, adding virtual patients, and increasing the severity of their injuries. The system is also able to make patient injuries dynamic so, for example, a first responder is required to act fast enough to prevent a virtual patient from experiencing fatal hemorrhage. To date, all of the features of First *VR*esponder have not been used for research, but these features hold promise for answering questions about triage effectiveness.

Another important area for future investigation is the cost-effectiveness and scalability of VR training programs. While VR technology has become more accessible, it is essential to evaluate the long-term costs and benefits of implementing such training on a larger scale. Studies could compare the costs of VR training with traditional methods, considering factors such as equipment, maintenance, and the potential for reducing the need for live drills. Additionally, research could explore how VR training can be scaled to accommodate large numbers of trainees across different regions and institutions. For example, recent work has shown the potential of artificial intelligence technology to conduct triage with some degree of success.^24^ These artificial intelligence agents could potentially be integrated into the VR environment to provide guidance and feedback during and after the training session, scaling the ability to train many trainees.

Finally, future research should continue to focus on comparing the effectiveness of the SALT triage algorithm to other established triage algorithms, such as START, JumpSTART, and Tactical Combat Casualty Care (TCCC) within the same standardized VR environment or using new VR scenarios. These comparisons could provide valuable insights into the strengths and weaknesses of each algorithm and help determine which is most effective in various scenarios. By using a standard and controlled VR environment, researchers can ensure that the comparisons are fair and free of bias.

## Conclusion

This study supports the use of VR to assess and train emergency responders in MCI triage and hemorrhage control using the SALT algorithm. Simulation with VR effectively distinguished performance by experience level and showed that even brief just-in-time SALT training enabled acceptable performance among medical trainees. These findings support the integration of VR into disaster preparedness curricula and lay the groundwork for future innovations in adaptive, scalable training platforms.

## Supporting information

Kman et al. supplementary material Kman et al. supplementary material

## Supplementary Material

To view supplementary material for this article, please visit https://doi.org/10.1017/S1049023X25101349
