# Supplementary material for: Virtual Reality Simulation for Assessment of Hemorrhage Control and SALT Triage Performance: A Comparison of Prehospital to In-Hospital Emergency Responders
Source: Prehosp Disaster Med. 2025 Aug;40(4):191–8. doi: 10.1017/S1049023X25101349 (PMC12809204; doi:10.1017/S1049023X25101349)
Supplement: Kman et al. supplementary material [file S1049023X25101349sup001.docx]

| Triage performance |  |
| --- | --- |
| Measure | Metric Definition |
| SALT Adherence: Execution of Global Sort Commands recommended by the SALT Triage Protocol | Whether or not the individual participant executed both the Walk and Wave commands at the beginning of the encounter (Yes vs. No). This provides an indication that they understood the purpose of the global sort commands as a technique for improving triage efficiency. |
| SALT Sequence Adherence Performance | The proportion of responders that consistently evaluated patients in the sequence recommended by the SALT Triage protocol: still, able to wave, and able to walk (see Appendix 2 for patients and their prescribed order). |
| Triage Tag Accuracy | The percentage of correct triage tags applied divided by the total number required triage tags (one for each virtual patient). This metric was also calculated separately for each tag category. |
| Over-triage errors | The percentage of patients that were placed into a higher priority triage category than required |
| Under-triage errors | The percentage of patients that were placed into a lower priority triage category than required |
| Critical triage errors | The percentage of triage errors that were likely to lead to patient morbidity or mortality (ex. placed erroneously into dead or expectant category) |
| Triage efficiency |  |
| Measure | Metric Definition |
| Time to Triage Scene | The start time of the session (when the participant started the triage procedure) until the end time of the session (when the last patient was treated and tagged) |
| Hemorrhage Control Completion for all Life-threatening Bleeding | The percentage of responders that achieved hemorrhage control for all life-threatening bleeding for the scene |
| Hemorrhage Control for all Life-threatening Bleeding | Duration of time from the scene start time until the time that the last required life-threatening bleeding injury has been treated with hemorrhage control procedures *(when the last tourniquet or wound packing was applied)* |
| Hemorrhage Control per Patient | Duration of time from when the patient was first approached by the participant until the time hemorrhage treatment was applied (with a tourniquet or wound packing) |

Appendix 1: Triage efficiency and performance metrics

Appendix 2: Prescribed SALT Treatment Order

Key: Group 1: Can be seen first. Obvious life-threatening hemorrhage should be prioritized. No patients from groups 2 or 3 should be seen prior to group 1.

| Correct order of FIRST11 |  |  |  |  |  |  |  |  |
| --- | --- | --- | --- | --- | --- | --- | --- | --- |
| Patient | Injuries | Vitals | SALT | Priority | Required Life Saving Intervention | SORT Group | Group Based on Most Severe Injury | Included in Hemorrhage Control Metric |
| Gary_3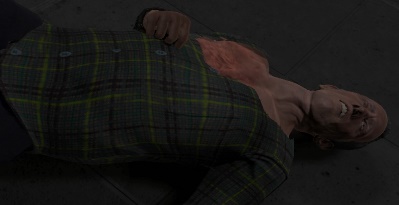 | R Collapsed Chest | Pulse fast  Resp. labored  Responds | Immediate | Still/Life Threat | AppliedDecompNeedle | 1 | 1 | No |
| Lily_2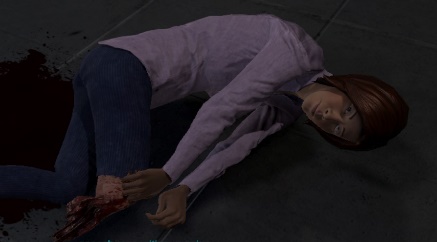 | R Shin Amputation | Pulse Fast  Resp. Fast  Responds  Waves | Immediate | Still/Life Threat | AppliedTourniquet | 1 | 1 | Yes |
| Bob_0  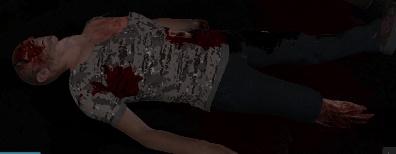 | -Face Shrapnel  -Collapsed Chest  -Stomach Puncture  -Amputations  -Thigh Laceration | Pulse absent  Resp. Absent  Responds No | Dead | Still/Life Threat | None | 1 | 1 | No |
| Gary_1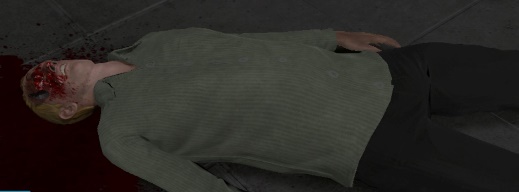 | Face Shrapnel | Pulse faint  Resp. none  Responds No | Expectant | Still/Life Threat | None | 1 | 1 | No |
| Mike_5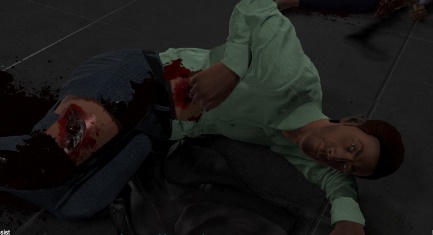 | -R Side Puncture  -R Thigh Laceration | Pulse Fast  Resp. Fast  Responds  Waves | Immediate | Still/Life Threat | -AppliedPackingGauze  -AppliedTourniquet | 1 | 1 | Yes |
| Lily_4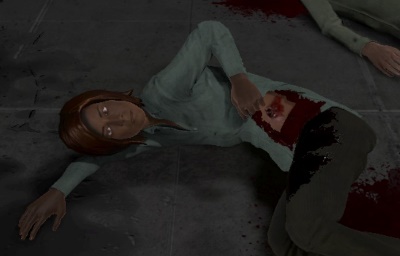 | L Side Puncture | Pulse fast  Resp. Normal  Waves | Immediate | Still/Life Threat | AppliedPackingGauze | 1 | 1 | Yes |
| Mike_7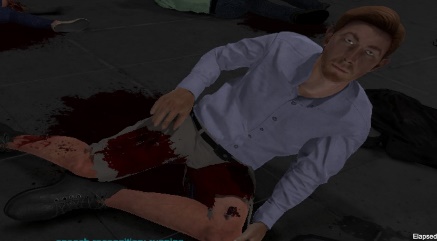 | -L Thigh Puncture  -Calf Laceration | Pulse Fast  Resp. Normal  Responds  Waves | Delayed | Still/Life Threat | -AppliedTourniquet  -AppliedDressingGauze (Optional) | 1 | 1 | Yes |
| Gloria_6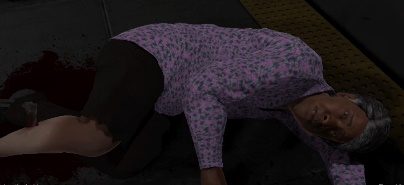 | Calf Shrapnel | Pulse Normal  Resp. Normal  Responds  Waves | Delayed | Wave | AppliedDressingGauze (optional) | 2 | 2 | No |
| Bob_9 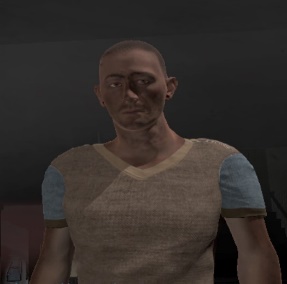 | Ear Bleed | Pulse normal  Resp. Normal  Responds?  Walks  Waves | Minimal | Walk | None | 3 | 3 | No |
| Gloria_8 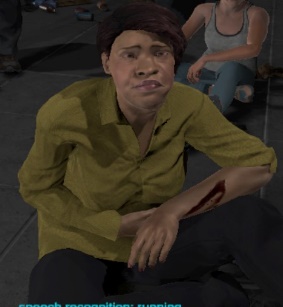 | L Forearm Laceration | Pulse normal  Resp. Normal  Responds  Walks  Waves | Minimal | Walk | AppliedDressingGauze (optional) | 3 | 3 | No |
| Helga_10 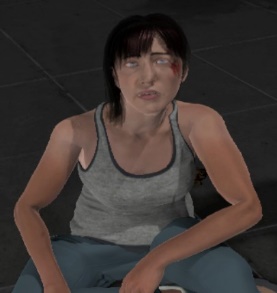 | Forehead Scrape | Pulse normal  Resp. Normal  Responds  Walks  Waves | Minimal | Walk | None | 3 | 3 | No |
